# Selection of Fitness Criteria for Learning Interpretable PDE Solutions via Symbolic Regression

**DOI:** 10.69997/sct.199083

**Published:** 2025-06-27

**Authors:** Benjamin G. Cohen, Burcu Beykal, George M. Bollas

**Affiliations:** a University of Connecticut, Department of Chemical and Biomolecular Engineering, Storrs, CT, USA; b University of Connecticut, Pratt & Whitney Institute for Advanced Systems Engineering, Storrs, CT, USA; c University of Connecticut, Center for Clean Energy Engineering, Storrs, CT, USA

**Keywords:** Genetic Algorithm, Machine Learning, Algorithms, Artificial Intelligence, Modelling

## Abstract

Physics-Informed Symbolic Regression (PISR) offers a pathway to discover human-interpretable solutions to partial differential equations (PDEs). This work investigates three fitness metrics within a PISR framework: PDE fitness, Bayesian Information Criterion (BIC), and a fitness metric proportional to the probability of a model given the data. Through experiments with Laplace’s equation, Burgers’ equation, and a nonlinear wave equation, we demonstrate that incorporating information theoretic criteria like BIC can yield higher fidelity models while maintaining interpretability. Our results show that BIC-based PISR achieved the best performance, identifying an exact solution to Laplace’s equation and finding solutions with $R^{2}$-values of 0.998 for Burgers’ equation and 0.957 for the nonlinear wave equation. The inclusion of the Bayes D-optimality criterion in estimating model probability strongly constrained solution complexity, limiting models to 3-4 parameters and reducing accuracy. These findings suggest that a two-stage approach-using simpler complexity metrics during initial solution discovery followed by a post-hoc identifiability analysis may be optimal for discovering interpretable and mathematically identifiable PDE solutions.

## INTRODUCTION

Understanding dynamical system models, their solutions, and potential failures is critical, especially in safety, health, and security. Traditionally, symbolic models with identifiable parameters, input-output relationships that provide tractable logic, and physically meaningful terms have established trust in deployed models [1]. However, these techniques to establish trust are ineffective for machine-learned models. Consequently, while machine learning has motivated advancements in understanding black-box model decisions [2], the simplicity and clarity of concise symbolic expressions make them inherently more straightforward to understand than their deep learning counterparts.

Dynamical system models often take the form of differential equations (DEs). When properly built and used, DEs enable engineers and scientists to predict future or hidden states while also explaining the underlying phenomena which drive system dynamics. Thus, DE-based system models are crucial for making informed decisions in process design and control.

Because of their importance, significant effort has been dedicated to simplifying and solving systems of PDEs [3,4]. Various methods for DEs include identifying coordinate transformations to simplify dynamics [5], linearizing models for rapid prediction and control, and finding analytical solutions to replace differential equations with algebraic relationships where possible. Depending on the DE and its application, different methods may be preferred. However, among the most challenging DEs to manage are those that include partial differential equations (PDEs).

Prominent in fluid flow, chemical reactions, and biological systems [6], PDEs can exhibit complex dynamics that make identifying solutions difficult. Common approaches for discovering PDE solutions are challenging to implement and require highly specialized skills and knowledge. Often PDE models can be linearized to leverage linear PDE theory, and Taylor series expansions can be used to find solutions via perturbation theory or finite differences among other methods. However, these solution paths are subject to strict convergence and stability conditions that are difficult to meet.

Alternatives to traditional methods are emerging from the field of machine learning [7]. Large foundation models for physics can help predict dynamics across various domains. Focusing on smaller problems, tools like DeepONets can learn neural operators that map inputs to outputs [8], while physics-informed machine learning methods can learn solutions to PDEs with known initial/boundary conditions [9, 10]. Although many of these tools are changing the way PDEs are solved, they often fall into the same pitfalls as other use cases of machine learning: the solutions they produce lack interpretability.

One exception to the lack of interpretability in machine learning for PDEs is physics-informed symbolic regression (PISR) [11, 12]. PISR leverages symbolic regression (SR) to produce solutions to PDEs in symbolic form. Previous works have shown that PISR can learn exact analytical solutions to PDEs when they exist and provide good approximations with physically meaningful terms when analytical solutions do not exist [11, 12]. Therefore, as an approach to simplify PDEs into explicit algebraic relationships, PISR is particularly attractive when model trust is important.

An important aspect of building model trust is to ensure that all model parameters are mathematically identifiable [1]. In this work, we explore various information-theoretic criteria as fitness metrics within PISR, beyond the metrics used in [11, 12], to learn interpretable solutions to PDEs that are simple, and concise. We examine the effect of the fitness criterion (loss function) within PISR on the discovered solutions through three examples: Laplace’s equation, Burgers’ equation, and a nonlinear wave equation.

The remainder of this paper is structured as follows: First, the PISR method and motivating examples will be introduced along with three fitness criteria. Following the methods, the results of our experiments are presented and briefly discussed. Finally, the paper concludes with a summary of the results and findings.

## Methods

The goal of PISR is to discover an explicit mathematical relationship that provides tractable logic from coordinate inputs to state outputs, ensuring consistency with a known PDE. This is achieved using symbolic regression. As the symbolic regressor generates different expressions, their derivatives are determined using automatic differentiation and evaluated against the known PDE. A solution with good fitness will have derivatives that satisfy the PDE, while those of an unfit solution are unlike to agree with that PDE. The symbolic regressor can take advantage of different fitness metrics to search for an optimal solution to a known PDE.

### Symbolic Regression

In this work, genetic programming (GP) is used to perform the symbolic regression. GP is an evolutionary algorithm that operates on a population of mathematical expressions, represented as binary expression trees, to explore the space of feasible expressions. Each individual in the population is constructed using an argument set and a primitive set. The argument set includes all arguments for the final model, while the primitive set is comprised of all mathematical operations that can appear in the model.

Genetic operators, along with evolutionary pressure from the selected fitness criterion and a tournament selection, iteratively evolve a population of expressions towards optimal fitness. Tournament selection is implemented using the selTournament method from the opensource evolutionary algorithm framework DEAP [13]. The genetic operators used can be categorized into crossover and mutation. In each generation, the population selected for offspring generation through tournament selection is subjected to these operators.

Crossover, an exploitative process, shuffles subexpressions from two individuals to create two offspring. The crossover operator used here is DEAP’s cxOnePoint [13]. Complementary to crossover, mutation is an exploratory process that makes random changes to a single individual to generate one offspring. Each expression used to generate offspring is operated on by one of four operators each generation: mutShrink, mutInsert, mutNodeReplacement, and mutUniform from DEAP’s library [13]. This combination of mutation operators helps explore the symbol space while reducing bloat, or the tendency of symbolic regression to evolve overly complicated expressions.

Once the population of offspring is generated using genetic operations, they are added to the total population along with a hall of fame that is made of the fittest individuals ever evolved. To promote diversity, some individuals are randomly removed. Before the next tournament selection can begin, fitness values must be calculated for any new individuals.

### Fitness Metrics

Fitness metrics are crucial in ensuring that the evolutionary pressure from the tournament selection process eliminates unfit individuals. However, different fitness measures may yield different solutions for the same PDE. Therefore, it is important to investigate the effect of different fitness criteria on the solutions learned for PDEs using PISR. Here, we introduce three fitness criteria used in the discovery of solutions to PDEs in this work.

### PDE Fitness

The first fitness criterion is the simplest PISR fitness metric, ${ℒ}_{PDE}$, which is defined in Equation 1. $N_{C}$ represents the number of collocation points at which the nonlinear PDE $F\left(x,u(x),D^{\alpha^{1}}u(x),D^{\alpha^{2}}u(x),\ldots ,D^{\alpha^{m}}u(x)\right)=0$, written using multi-index notation, is evaluated in the n-dimensional domain $X\subseteq R^{n}$. The variables $\lambda_{0}$, $\lambda_{j}$, $N_{BCs}$, $N_{BC_{j}}$, and $\Gamma_{j}$ respectively represent hyperparameters for the PDE, hyperparameters for each boundary condition, the number of boundary conditions, the number of collocation points on the $j^{\text{th}}$ boundary condition, and the $j^{\text{th}}$ boundary condition. The goal of ${ℒ}_{PDE}$ is to capture how well an expression represents a PDE and its initial and boundary conditions.


(1) $${ℒ}_{PDE}=\lambda_{0}\sum_{i=1}^{N_{C}}{F(x_{i},u(x_{i}),\ldots )}^{2}+\sum_{j=1}^{N_{BCs}}\lambda_{j}\sum_{k=1}^{N_{BC_{j}}}\Gamma_{j}{\left(x_{k},u(x_{k}),\ldots \right)}^{2}$$

To calculate ${ℒ}_{PDE}$, the derivatives of the expression being evaluated must be determined. In this work, we used the automatic differentiation tool CasADi [14] to obtain exact derivatives quickly and efficiently. Next, those derivatives must be used to evaluate F at the $N_{C}$ collocation points and each $\Gamma_{j}$ at the $N_{BC_{j}}$ collocation points. Table 1 shows a symbolic evaluation of $F=u_{t}-\alpha u_{xx}=0$ with constant α for two different expressions, demonstrating how ${ℒ}_{PDE}$ can be determined.

### Bayesian Information Criterion

A limitation of ${ℒ}_{PDE}$ is that it only evaluates how well the proposed expressions satisfy the PDE and boundary conditions at the collocation points. While this is crucial for obtaining accurate solutions to PDEs, it is also important to consider the complexity of the relationships between the input coordinates and the state output. ${ℒ}_{PDE}$ does not provide safeguards against excessing model complexity, meaning the symbolic regressor can quickly suffer from expression bloat and learn overly complicated expressions that are difficult to interpret and understand.

One potential remedy to this limitation is to use the Bayesian Information Criterion (BIC) [15]. The BIC is an information-theoretic criterion that helps mitigate model complexity by balancing the fitness of the expression in terms of both its agreement with the PDE and its complexity. Equation 2 calculates the BIC, where $N_{points}$ represents the weighted number of collocation points used (given by $\lambda_{0}N_{C}+\sum_{j=1}^{N_{BCs}}\lambda_{j}N_{BC_{j}}$), and k denotes the model node complexity, which is determined by counting the number of nodes in the expression tree.


(2) $${ℒ}_{\text{BIC}}=N_{points}\;\log\;{ℒ}_{PDE}+k\;\log\;N_{points}$$

### Probability of Solution

The BIC is often a powerful criterion for identifying concise and descriptive expressions in symbolic regression. However, in many cases, simply balancing model complexity and fit does not guarantee that the model is identifiable. To ensure that each model parameter is mathematically identifiable based on the information provided, an additional term must be used.

A strong candidate for this additional term is the Bayes D-optimal criterion, $\Psi_{D}$ [16]. $\Psi_{D}$ is an information-theoretic criterion that uses the determinant, denoted as ∣⋅∣, of the Fisher Information Matrix (FIM) to give insight into the precision of parameter estimates. It can help determine if a model is identifiable, meaning that there exits a unique parameter set defining the optimal solution [16]. $\Psi_{D}$ also appears in the derivation of the BIC but is omitted in its final form due to asymptotic assumptions.

The FIM can be calculated using the sensitivity matrix $Q=\nabla_{\theta }{ℒ}_{PDE}$, where θ is the vector of parameters proposed by the symbolic regressor. Typically, the sensitivity matrix is calculated with a variance term to account for noise in the data. However, in PISR, since there is no data, this variance term is omitted, implicitly assuming a variance of 1. With Q defined, we can now introduce the third fitness criterion, ${ℒ}_{BIC,\Psi_{D}}$, as shown in Equation 3. This criterion is proportional to approximate probability of the PDE given the model m at the maximum likelihood estimator $\hat{\theta }$, p(F∣m), which is derived using the same process as the derivation of the BIC.


(3) $${ℒ}_{BIC,\Psi_{D}}={ℒ}_{BIC}-\log|Q^{T}Q|$$

### Test Cases

With the fitness criteria established, the performance of PISR using each criterion must be assessed. This evaluation was conducted using three two-dimensional second-order PDEs. For each criterion and PDE pair, PISR was run 50 times, with support from a 30 × 30 grid of collocation points. The PDEs and boundary conditions were evaluated on this grid, and each run produced a hall of fame containing the 10 most fit evolved solutions. The experiments were executed on an Intel^®^ Xeon^®^ W7-2495X processor across 10 threads.

Solution evaluation was carried out on a 50 × 50 gird of points, where the true solution at each point was computed either using an exact analytical solution or a numerical method. The symbolically regressed models were compared using three metrics: mean-squared error (MSE) against the ground truth, the $R^{2}$-value against the ground truth, and model parameter complexity, $N_{p}$, defined as the number of parameters in the model.

### Laplace’s Equation

The first PDE investigated was Laplace’s equation, a second-order linear PDE with an exact analytical solution for the boundary conditions specified in Equation 4. Laplace’s equation is a fundamental PDE in process systems engineering, as it models many steady-state phenomena such as heat transfer and diffusion. The domain for this PDE, as explored in this study, was (x,y)∈[0,1]×[0,1]. The primitives used to discover solutions to Laplace’s equation were {+,−,exp,sin,sinh}, with arguments {x,y}. The tuning parameters for ${ℒ}_{PDE}$ were selected as λ=[0.001,0.8,0.8,80,0.8].


(4) $$\;u_{xx}+u_{yy}=0\;\;u(x,y)=0\;\;\;\text{when}\;x=0\;\;u(x,y)=0\;\;\;\text{when}\;x=1\;u(x,y)=\sin(2\pi x)\;\text{when}\;y=0\;\;u(x,y)=0\;\;\;\text{when}\;y=1$$

### Burgers’ Equation

The second PDE explored in this work was Burgers’ equation, which is often used to describe one-dimensional viscous fluid flow. It is a quasi-linear second-order PDE in two dimensions and does not have an exact strong form analytical solution. The domain considered in this work was (x,t)∈[0,10]×[0,10]. The primitives used to discover solutions to Burgers’ equation were $\left\{+,\times ,\text{exp},\sin,\sin h,u_{diff}\right\}$, where $u_{diff}$ is the solution to the diffusion equation with the same initial and boundary conditions in Equation 5, solved using eigenfunction expansion. For more details on the method of the primitive set selection see [12]. The arguments used were {x,t}, and the tuning parameters for ${ℒ}_{PDE}$ were λ=[1,0.8,0.8,0.8].


(5) $$\;u_{t}-0.1u_{xx}-0.2{uu}_{x}=0\;u(x,0)=\text{exp}-0.2{\left(x-5\right)}^{2}\;\;\text{when}\;t=0\;\;u_{x}(0,t)=0\;\;\;\text{when}\;x=0\;\;u_{x}(10,t)=0\;\;\;\text{when}\;x=10$$

### Nonlinear Wave Equation

The third and final PDE explored in this work was the nonlinear wave equation, which is commonly used to model waves in shallow water. It is a nonlinear second-order PDE in two dimensions and, like Burgers’ equation, does not have an exact analytical solution. The domain considered in this work was (x,t)∈[0,1]×[0,1]. The primitives used to discover solutions to the nonlinear wave equation were $\left\{+,\times ,\text{exp},\sin,\sinh,u_{\text{linwave}}\right\}$, where $u_{linwave}$ is the analytical solution to the linear wave equation with the same initial and boundary conditions in Equation 6. The arguments used were {x,t}, and the tuning parameters for ${ℒ}_{PDE}$ were λ=[0.1,0.8,0.8,80].


(6) $$\;u_{tt}-u^{2}u_{xx}=0\;u(x,0)=\frac{1}{2}\sin(3\pi x)\;\text{when}\;t=0\;\;u(0,t)=0\;\;\;\text{when}\;x=0\;\;u(1,t)=0\;\;\;\text{when}\;x=1$$

## RESULTS AND DISCUSSION

The results of our experiments demonstrate that the selection of fitness criterion can significantly influence the solutions discovered using PISR. When model complexity is considered during exploration, PISR tends to return models with fewer parameters. Moreover, when PISR produces models with more parameters, those trained with complexity considerations align better with the solution to the PDE compared to those trained without considering complexity. When a metric of identifiability, or the ability for all parameter values in a model to be uniquely identified given the available information, is incorporated into the fitness criterion, PISR is more likely to return models with only a few parameters and those models tend to perform as well or worse than models learned using other fitness metrics.

### Results: Laplace’s Equation

These relationships between fitness metrics and the discovered solutions are particularly clear for Laplace’s equation. Laplace’s equation, with the boundary conditions shown in Equation 4, has an exact analytical solution, allowing the learned solutions to be compared symbolically with the exact solution. PISR successfully learned the exact solution to Laplace’s equation using ${ℒ}_{PDE}$ and ${ℒ}_{PBIC}$ as fitness metrics, but not when usings ${ℒ}_{BIC,\Psi_{D}}$.

**Figure 1:**
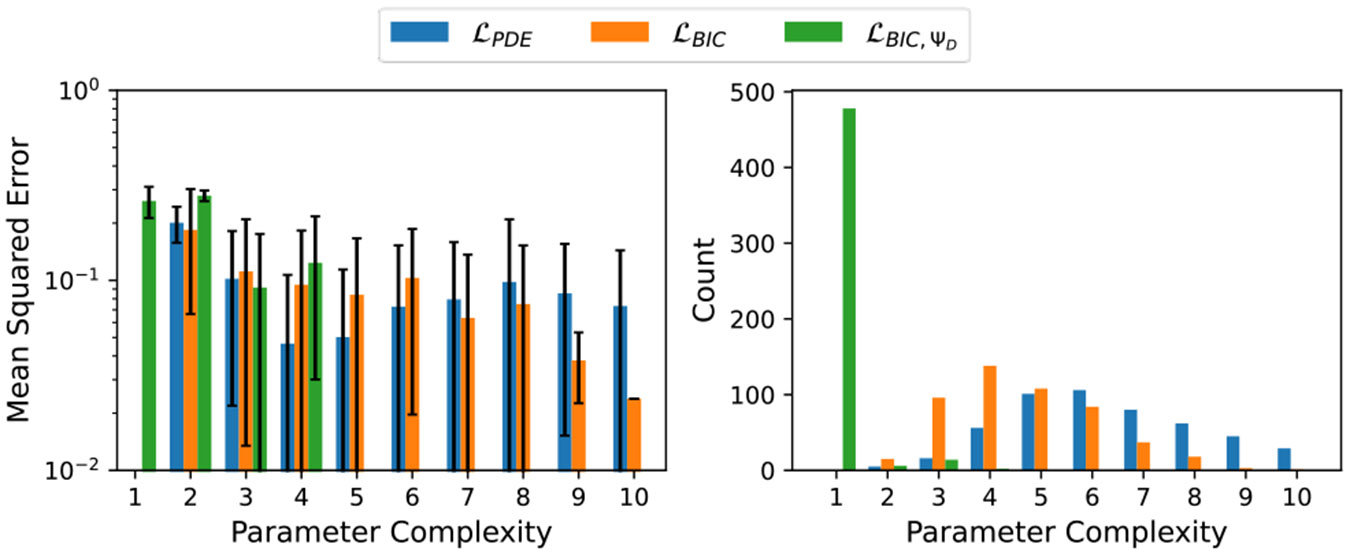
Left: Average MSE values per model parameter complexity for Laplace's equation solution discovery. Right: Count of learned models with different parameter complexities.

Although PISR did learn exact analytical solutions to Laplace’s equation, on average, the models discovered across the 50 runs for each fitness criteria did not perfectly match an analytical solution. Figure 1 illustrates the average MSE values at different model parameter complexities. This figure also shows the number of models returned at each parameter complexity for the different fitness metrics.

The results show that the selection of fitness criteria has little impact on the quality of the solutions when models have only a few parameters. However, the fitness criteria did affect the likelihood of a learned model have a small number of parameters, with stricter definitions of complexity leading to more models with fewer parameters. As the number of parameters in a model increases, ${ℒ}_{BIC}$ tends to yield the best solutions on average. Notably, no model learned using ${ℒ}_{BIC,\Psi_{D}}$ had more than 4 parameters.

While limiting model complexity may be advantageous for building models that are easy to interpret, it can also impede the discovery of better models. By including the $\Psi_{D}$ term in the fitness, the symbol space is constrained to consider only expressions that are mathematically identifiable. This constraint may make it more difficult to discover models that fit the underlying PDE well, as it restricts access to certain regions of the symbol space in which useful intermediate expressions may lie.

### Results: Burgers’ Equation

The results from the Burgers’ equation experiments tell a similar story to those from Laplace’s equation. The best-learned models using each of the three fitness criteria are shown in Table 2. Since Burgers’ equation does not have an exact analytical solution, all metrics of agreement with the ground truth were compared to the solution computed using the method of lines.

As in the previous experiment, ${ℒ}_{PDE}$ and ${ℒ}_{BIC}$ produced better solutions than ${ℒ}_{BIC,\Psi_{D}}$. The best model was discovered using ${ℒ}_{BIC}$. Furthermore, the best solutions learned using all three fitness criteria have similar structures shown in Table 2.

**Figure 2:**
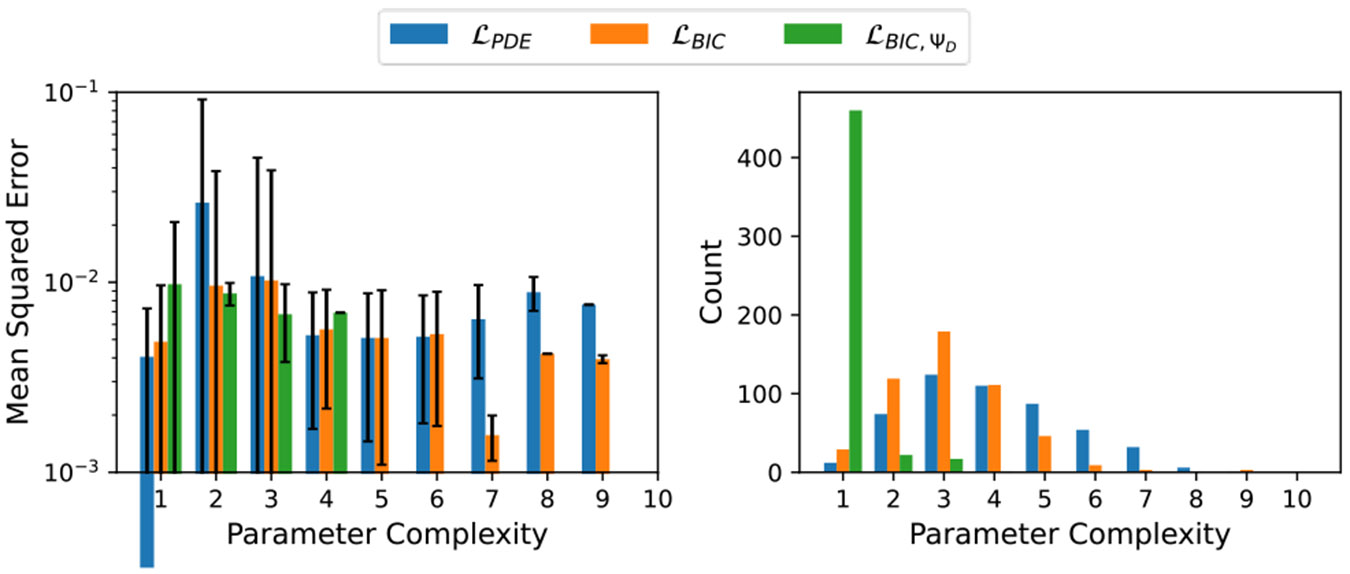
Left: Average MSE values per parameter complexity Burgers’ equation solution discovery. Right: Count of learned models with different parameter complexities.

To better understand the average performance of the discovered solutions for Burgers’ equation, Figure 2 displays the average MSE values at different model parameter complexities, along with the number of models returned at each parameter complexity for the different fitness metrics. Analysis of these plot reveal the same trends as those discussed for Figure 1.

### Results: Nonlinear Wave Equation

The third PDE explored was the nonlinear wave equation, which proved more challenging to solve using PISR than the other two PDEs. This increased difficulty in solution discovery is reflected in the learned models, which . Table 3 presents the model parameter complexities, $N_{p}$, node complexities, k, as well as the MSE and $R^{2}$-values of the best solutions discovered using the three fitness metrics. These complexity measures were chosen because the learned solutions are long and difficult to interpret, although still have fewer parameters than solutions learned using neural networks.

These solutions are significantly more (node) complex than those learned for the previous two PDEs. Nevertheless, all three best solutions use fewer than 10 parameters, and PISR with ${ℒ}_{PDE}$ and ${ℒ}_{BIC}$ returned models with $R^{2}$-values greater than 0.9. With more time, effort, and hyperparameter optimization, it is possible even better symbolic solutions exist within the explored symbol space. Once again, PISR using ${ℒ}_{BIC}$ yielded the best model among the fitness metrics considered.

**Figure 3:**
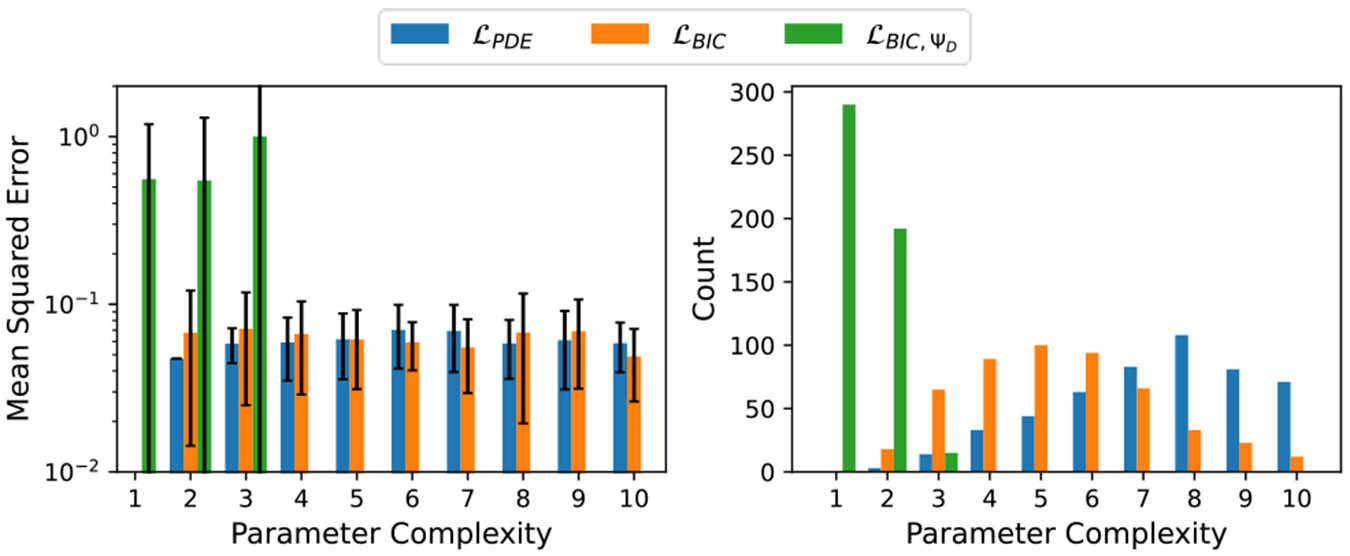
Left: Average MSE values per parameter complexity of Wave equation solution discovery. Right: Count of learned models with different parameter complexities.

Figure 3 shows the average MSE values at different model parameter complexities and the number of models returned at each model parameter complexity for the three fitness criteria. Consistent with the results from the other two cases, the results shown in the right-side plot of Figure 3 follow a similar pattern with respect to the number of models learned at each parameter complexity. However, unlike the other two cases, solutions learned using ${ℒ}_{BIC}$ do not, on average, perform better than those learned using ${ℒ}_{PDE}$ as the parameter complexity increases. At low parameter complexity, solutions learned using ${ℒ}_{BIC,\Psi_{D}}$ perform worse than those discovered using other fitness metrics.

## CONCLUSIONS

PISR is an approach for learning symbolic, interpretable solutions to PDEs. Its efficacy varies depending on the type of PDE and it can struggle to find a balance between interpretability and accuracy in representations to solutions of complex PDEs. Its performance can be influenced by the selection of a fitness criterion. In this work, we demonstrate that using an information-theoretic fitness criterion can help learn higher-fidelity solutions to PDEs. Penalizing model complexity can promote simpler, more easily interpreted solutions. However, using metrics that incorporate model identifiability can constrain the symbol space, potentially hindering solution discovery.

This tension between model fit and simplicity is difficult to balance. While mathematically identifiable solutions are often desired, our findings suggest it may be more effective to use symbolic regression with less stringent metrics, such as parameter or node complexity, and to access mathematical identifiability post hoc, only after candidate solutions have been discovered.

## Figures and Tables

**Table 1:** Example of symbolic evaluation of $F=u_{t}-\alpha u_{xx}$ for different expressions when α is a constant.

u	$u_{t}$	$u_{xx}$	F
$t-\alpha x^{3}$	1	−6αx	$1+6\alpha^{2}x$
$\text{exp}(-\alpha \omega^{2}t)\;\sin(\omega x)$	$-\alpha \omega^{2}u$	$-\omega^{2}u$	0

**Table 2:** Best models learned for Burgers’ equation using different fitness criteria. The MSE and $R^{2}$-values are calculated using the solution calculated using the method of lines as the ground truth.

Fitness	Model	$\mathrm{MSE}\times 10^{4}$	$R^{2}$
${ℒ}_{PDE}$	$u_{diff}[x-0.189t\cdot u_{diff}(x,17.317),t]$	3.850	0.996
${ℒ}_{BIC}$	$u_{diff}[x-0.167t\cdot u_{diff}(x-0.066x,t),t]$	1.796	0.998
${ℒ}_{BIC,\Psi_{D}}$	$u_{diff}[x-0.174t\cdot u_{diff}(x,x+t),t]$	4.466	0.995

**Table 3:** Characteristics of best models learned for Burgers’ equation using different fitness criteria. The MSE and R2-values are calculated using the solution calculated using the method of lines as the ground truth.

Fitness	$N_{p}$	k	MSE	$R^{2}$
${ℒ}_{PDE}$	5	40	0.020	0.941
${ℒ}_{BIC}$	5	27	0.015	0.957
${ℒ}_{BIC,\Psi_{D}}$	3	19	0.125	0.633
